# Recent Land Use Change to Agriculture in the U.S. Lake States: Impacts on Cellulosic Biomass Potential and Natural Lands

**DOI:** 10.1371/journal.pone.0148566

**Published:** 2016-02-11

**Authors:** David J. Mladenoff, Ritvik Sahajpal, Christopher P. Johnson, David E. Rothstein

**Affiliations:** 1 Department of Forest and Wildlife Ecology, University of Wisconsin-Madison, 1630 Linden Drive, Madison, WI, 53706, United States of America; 2 Department of Forestry, Michigan State University, 126 Natural Resource Building, East Lansing, MI, 48824, United States of America; DOE Pacific Northwest National Laboratory, UNITED STATES

## Abstract

Perennial cellulosic feedstocks may have potential to reduce life-cycle greenhouse gas (GHG) emissions by offsetting fossil fuels. However, this potential depends on meeting a number of important criteria involving land cover change, including avoiding displacement of agricultural production, not reducing uncultivated natural lands that provide biodiversity habitat and other valued ecosystem services, and avoiding the carbon debt (the amount of time needed to repay the initial carbon loss) that accompanies displacing natural lands. It is unclear whether recent agricultural expansion in the United States competes with lands potentially suited for bioenergy feedstocks. Here, we evaluate how recent land cover change (2008–2013) has affected the availability of lands potentially suited for bioenergy feedstock production in the U.S. Lake States (Minnesota, Wisconsin, Michigan) and its impact on other natural ecosystems. The region is potentially well suited for a diversity of bioenergy production systems, both grasses and woody biomass, due to the widespread forest economy in the north and agricultural economy in the south. Based on remotely-sensed data, our results show that between 2008 and 2013, 836,000 ha of non-agricultural open lands were already converted to agricultural uses in the Lake States, a loss of nearly 37%. The greatest relative changes occurred in the southern half that includes some of the most diverse cultivable lands in the country. We use transition diagrams to reveal gross changes that can be obscured if only net change is considered. Our results indicate that expansion of row crops (corn, soybean) was responsible for the majority of open land loss. Even if recently lost open lands were brought into perennial feedstock production, there would a substantial carbon debt. This reduction in open land availability for biomass production is closing the window of opportunity to establish a sustainable cellulosic feedstock economy in the Lake States as mandated by current Federal policy, incurring a substantial GHG debt, and displacing a range of other natural ecosystems and their services.

## Introduction

Bioenergy derived from perennial cellulosic energy crops, including woody biomass, may have the potential to reduce life-cycle GHG emissions while providing better wildlife habitat and a range of ecosystem services that are not present, and often diminished, in today’s corn-grain ethanol production systems [1–9]. Currently, row crops like corn and soybean dominate biofuel production due to the absence of proven cost-effective and scalable technologies to produce biofuels from cellulosic feedstocks. However, recent research may improve the viability of cellulosic feedstocks [10], and successful operation of a number of pilot cellulosic biorefinery projects in the U.S. has improved the prospects of biofuels produced from these feedstocks [11]. The U.S. bioenergy production targets are ambitious: 136 billion liters per year by 2022, of which 76 billion liters are to be produced from second generation cellulosic energy crops [12]. However, reductions in life-cycle GHG emissions from perennial cellulosics are not guaranteed, nor are gains in ecosystem services and habitat quality after establishment of energy feedstocks.

GHG reductions can be quickly negated and emissions increased under a number of circumstances, including the displacement of agricultural food, feed, forest wood or fiber production. As well, significant carbon debt can be incurred, particularly by initiating cultivation of productive natural lands rich in carbon and nitrogen [13–17]. Lands that are low in carbon and nitrogen are generally not optimal for agriculture are often called ‘marginal lands’, and have been widely targeted in recent years as the most sustainable locations for the establishment of perennial cellulosic energy crops [18–21], often due to the assumed lack of competition with agriculture [22–23]. Although these ecosystems are marginal for agriculture, they have other existing values, including habitat, watershed protection and sequestered carbon. Converting these to bioenergy production can have a net benefit to habitat and other ecosystem services if they replace degraded lands that have less valued habitats or existing agriculture.

The actual availability of marginal lands for biofuel production, now and into the future, is questionable, as the United States is in a time of unparalleled expansion of agriculture [24–25]. This, in part, has resulted in the conversion of non-agricultural marginal lands—the same lands thought to be optimal for sustainable perennial cellulosic feedstock production—to agricultural cropping [24]. To date, there is a lack of information detailing the extent of agricultural uses on marginal lands and the ways in which the use of marginal lands are changing. Gelfand et al. [23] used a high resolution 2008 land cover/use dataset to identify land optimal for bioenergy feedstock establishment in the north-central United States based on all non-forested land covers on marginal sites. The present study differs from their approach by separately analyzing agricultural lands (e.g., corn and soybeans) and non-agricultural open lands (e.g., grasslands and shrublands). Wright and Wimberly [24] showed that corn and soybeans expanded onto non-agricultural marginal grasslands between 2006 and 2011 in the western and northern Corn Belt (North Dakota, South Dakota, Nebraska, Iowa, and Minnesota). At a national scale, Lark et al. [25], documented cropland intensification and extensification between 2008 and 2012. We build on their approach by providing data on other land covers and demonstrating the complex land cover change patterns [26] that are occurring in the Lake States due to agricultural expansion, a region of both agricultural and forestry land use. Here, we provide a full analysis of direct land cover change between 2008 and 2013 in the Lake States (Michigan, MI; Minnesota, MN; and Wisconsin, WI), USA and identify the site-quality classes onto which these changes are occurring. The Lake States are considered in this study for two reasons: First, they have been suggested to offer significant potential for bioenergy production from both short-rotation woody crops (SRWCs) in the largely forested north as well as non-woody perennial cellulosics in the south [27]. Second, past and anticipated land cover change [28–29] has the potential to influence the direction and magnitude of GHG emissions from bioenergy systems.

It is common for agricultural lands to move into retirement, and retired lands to come back into production, for example with the Conservation Reserve Program (CRP) [30–31]. The area of land enrolled in CRP peaked in 2007, and has been declining since, and growing evidence shows that other lands are also being converted to agriculture [24–25]. This is likely driven by current mandates for corn ethanol production (USEPA 2010) and resulting in higher market prices for corn [32]; the effect of changes made in federal law in 1995 that allowed lands formerly protected under “sodbuster” and “swampbuster” rules, and therefore excluded from Federal crop protection insurance, be covered by insurance [33]. The former risk of cultivating these agriculturally marginal lands was removed. The 2014 federal Farm Bill (Agricultural Act of 2014) reversed some of these effects with a “sodsaver” protection, but only for the Northern Great Plains, affecting only Minnesota in our study region. Under current ethanol market conditions, these changes still drive conversion of formerly unsuitable lands to agriculture outside the six northern Great Plains states [34]. Further, the U.S. Renewable Fuels Standard [35] also requires that U.S. EPA monitor that renewable fuels are not coming from lands converted to agriculture after 2007 to address these concerns of land use change and their associated emissions. But this decrease in CRP and other non-agricultural lands is continuing [24, 36], and is likely having two major environmental effects in the Lake States: First, as non-agricultural lands are converted to agricultural uses, the area of land that could be put into perennial cellulosic plantations without displacing food, feed and forest wood and fiber decreases, thereby lowering the GHG-reduction potentials for the region. Second, converting native ecosystems to agriculture has a number of negative environmental effects, including losses of habitat and biodiversity [7, 37], degradation of water quality and quantity [1, 38, 39], and increases in GHG emissions [15, 40, 41], especially at initial cultivation [17] incurring a substantial GHG debt.

The exact extent and consequences of agricultural expansion that has occurred in the Lake States is unknown. Our basic question is, how have recent land cover patterns changed under current drivers, and how has this affected open, non-agricultural lands assumed available for cellulosic biomass production? Thus, the overall objectives of this study are threefold: First, we analyze how recent land cover change (2008 to 2013) has affected the area of agricultural and non-agricultural open lands on marginal, moderate, and productive site-quality classes. Second, we analyze more complex patterns of land cover change that are masked by simple analyses of only net change through the use of transition diagrams and matrices. Agriculture does not simply expand onto new lands, but crop rotations change, agricultural lands come out of production, and idle lands go into production [30]. Third, these analyses provide insight into the more detailed impact that land cover change is having in the region (e.g., increasing GHG emissions, loss of ecosystem services), and the ways in which such changes are creating or constraining opportunities for the expansion of perennial cellulosic feedstocks.

## Methods

### Study Region

**Fig 1:**
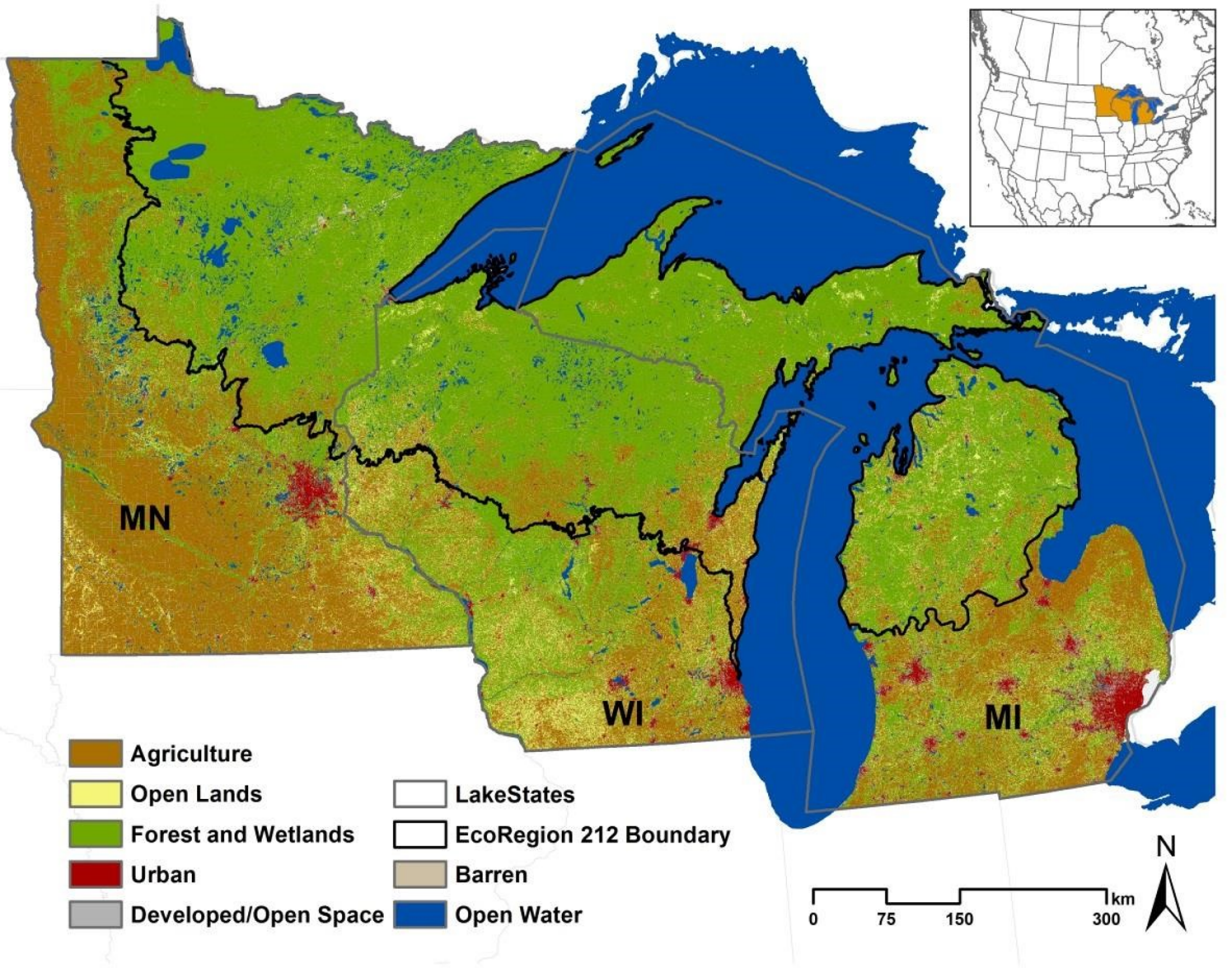
Land use/cover of the Lake States, USA (MN, Minnesota; WI, Wisconsin; MI, Michigan) derived from the 2012 Cropland Data Layer. Land-cover categories were simplified to depict the major categories analyzed here.

This analysis includes the Lake States, USA (Michigan, MI; Minnesota, MN; and Wisconsin, WI), which extend from approximately 49° 23’ in the north, to 41° 42’ in the south, -82° 25’ in the east and -97° 13’ in the west (Fig 1). MN is the largest of the three states, with an area of approximately 21.9 x10^6^ hectares, followed by MI (15.1x106 ha), and WI (14.5 x10^6^ ha). There are three Ecoregions in the Lake States: Ecoregion 212, the northern-most Laurentian Mixed Forest Province; Ecoregion 222, the Midwest Broadleaf Forest Province; and Ecoregion 251, the Prairie Parkland (temperate) Province (Fig A in S1 File) [42]. We grouped Ecoregions 222 and 251 (herein referred to collectively as “the south” or “southern region”) together to compare against Ecoregion 212 (herein referred to as “the north” or “northern region”), which accounts for approximately 50% of the total area of the Lake States (~ 25.78 x10^6^ ha). The northern region is dominated by forests and wetlands, thousands of lakes, and some grasslands, shrublands, and agriculture intermixed within the forest and wetland matrix. Agriculture dominates much of the southern areas, especially in southwestern MN (Ecoregion 251). The southern and central Lake States (Ecoregion 222) are largely a mix of agriculture and grasslands with some localized concentrations of forests and wetlands. WI has the most heterogeneous land cover in the Lake States, with some large areas of forests in the south and agriculture in the north.

The Lake States climate is temperate continental, but is moderated near the Great Lakes. In the south, average summer (June, July, August) high temperatures are approximately 26.6°C and winter (December, January, February) lows are approximately -10.2°C (Madison, WI; 1981 to 2010 average) [43]. In the north, average summer highs are approximately 23.9°C and average winter low temperatures are approximately -20.5°C (Ely, MN; 1981 to 2010 average) [43]. Generally, most of the annual precipitation falls during the growing season, except for localized lake-effect snow.

### Datasets

We used the U.S. Department of Agriculture (USDA) National Agricultural Statistics Service (NASS) Cropland Data Layer (CDL) to analyze land cover and land use changes in the region. We downloaded the CDL raster data for the WCB states from http://nassgeodata.gmu.edu/CropScape/ [44]. The CDL is a state-level, raster-formatted thematic land-cover map that is published annually. The rasters were acquired in Albers conical equal area projection, one file for each year for each state. Each thematic CDL raster has a set of unique identifiers which represent discrete features of crop cover type or in the absence of cropping, the land cover. CDL data for all three Lake States has been available since 2007, providing seven years of land cover information for analysis. While prior to 2007 CDL data includes metadata information on the level and extent of cloud contamination, starting in 2007, CDL data are provided with cloud-free coverage. The CDL is produced annually via supervised classification of remotely sensed images from a number of different sources including MODIS, Advanced Wide Field Sensor (AWiFS) and Landsat Thematic Mapper (TM) [45]. Training data for agricultural classes (corn, soybean etc.) are gathered through the USDA June Agricultural Survey and spatially-explicit, county-level records of farm-field boundaries maintained by the Farm Service Agency; training data for non-agricultural areas are gathered from the most recent US Geological Survey, National Elevation Dataset (NED), National Land Cover Dataset (NLCD) percent tree cover and percent impervious products [45].

The CDL classification accuracy of commodity crops (corn, soybean and wheat) is high (80–95%), while the accuracy of non-crop classes is generally lower, especially for grass and shrub classes [45, 46]. To account for the low accuracy of grassland and shrubland classes (CDL: #152-shrubland; #176-grass/pasture) these classes are combined to create a single ‘grassland’ or ‘open lands’ class [24, 46]. Here we also include CDL #61-fallow/idle cropland to create a single ‘Open Lands’ category that represents non-agricultural open lands (Table 1). We also combined forest and wetland classes into a single class (‘Forests/Wetlands’), all five developed/urban classes into a single class (‘Developed’), and created an ‘Other Agriculture’ class that contains all managed lands except for corn (CDL #1) and soybeans (CDL #5) (Table 1). The spatial resolution of the CDL is 56-m prior to the year 2010 and 30-m thereafter. Since it is generally accepted that a cell size finer than the coarsest input resolution will not produce more accurate data than the input, we used ArcGIS 10.2™ to resample all 30-m rasters to the coarser 56-m resolution, In order to minimize errors arising from spatial mismatch of raster cells from one year to next, we set the snap environment in ArcGIS 10.2™ to ensure the 30-m rasters overlaid the older 56-m rasters correctly.

**Table 1:** Breakdown of the individual Cropland Data Layer (CDL) classes that comprise the land groups used in this analysis.

	**CDL codes**
Open lands	61 Fallow/Idle Cropland, 152 Shrubland, 176 Grass/Pasture
Corn	1 Corn
Soybeans	5 Soybeans
Other agriculture	All others except water, barren and no data
Forests/Wetlands	63 Forest, 87 Wetlands, 141 Deciduous Forest, 142 Evergreen Forest, 143 Mixed Forest, 190 Woody Wetlands, 195 Herbaceous Wetlands
Developed	82 Developed, 121 Developed/Open Space, 122 Developed/Low Intensity, 123 Developed/Med Intensity, 124 Developed/High Intensity
**Site quality class**	**SSURGO land capability class (LCC)**
Productive	1, 2
Moderate	3, 4
Marginal	5, 6, 7

The CDL codes are represented by a unique identifier and the corresponding land cover name. Agricultural crops are broken down into three categories, and are highlighted in gray. Also provided are the Soil Survey Geographic (SSURGO) land capability class (LCC) values corresponding to each site quality class.

We used the USDA Soil Survey Geographic Dataset (SSURGO) Land Capability Class (LCC) to define site quality. The LCC is one of the most widely available and standardized measures of the relative suitability of soils to produce commodity crops in the US. It groups soils into one of eight classes based on potential suitability and risk of erosion or the presence of a limiting factor (e.g. hard pan, droughtiness, or flooding). Class 1 soils are of the highest quality and have no limiting factor. Classes 2–8 have a corresponding subclass that defines the most limiting factor: slope, climate, erosion, wetness. Class 8 soils can be comprised of rocky outcrops or quarries and are therefore not suitable for cultivation, with their only utility being in providing cover for wildlife or recreational uses. The native format of SSURGO data is polygon with corresponding spatial (shapefiles) and database counterparts. To utilize these data across the whole Lake States region and to match the CDL data, we created regional, 56-meter resolution raster files of LCC (Fig B in S1 File). There are a few gaps in the LCC data, including some entirely missing counties in MN, creating a slight difference in the land area based on CDL/LCC combined data and CDL-only data. Forest/wetland and water comprised over 90% of this land area.

We used the USGS Public Areas Database (PAD) Version 3 (GAP 2013, [47]) to determine which lands are in public holding. We assume that if cellulosic bioenergy crops become established in the Lake States, it will occur predominately on non-public lands [48]. We used this dataset to constrain our estimates of land potentially available for energy crops (see “Biophysical land identification” below).

### Biophysical Land Identification

To determine how agricultural expansion has changed the use of lands with varying site quality, we created six groups based on three site-quality classes (productive = LCC 1 and 2; moderate = LCC 3 and 4; marginal = LCC 5, 6, and 7) and two land groups from the CDL (open lands and agricultural lands) (Table 1). Using ArcGIS 10.2™, we combined the LCC and CDL datasets and then queried each of the six groups for the northern and southern regions of the Lake States. Selected lands in public holding (from the PAD dataset) were removed, and single pixels were filtered in an attempt to control some of the inherent error that is present in thematic maps and to present a more conservative estimate of selected lands (after [24]). Our choice of a single-pixel filtering approach is due to the heterogeneity in the Lake States landscape, compared to the more aggressive 5x5 majority filter utilized by Wright and Wimberly [24] in the generally more homogeneous western cornbelt. This querying and filtering was done using both the 2008 and 2013 CDL datasets in order to identify the effects of land cover change on the area of each land group. All non-public, pixel-filtered lands were grouped into one of six classes (productive, moderate, and marginal site-quality classes for each of agricultural and non-agricultural open lands) (Table 1) that represent a range of potential suitability and environmental impact gradients.

### Land Cover Change Analysis

To provide best-estimate values to the area of land in agricultural and non-agricultural classes potentially suited for SRWC plantations, we filtered raw data to remove areas in public holding using the Public Areas Database. We also filtered away single pixels that we believed were largely remnants of classification error.

To determine how land cover has changed in recent years, we analyzed the area and relative change of the major land groups described above between 2008 and 2013. Our choice of 2008 as the starting year for analysis avoided classification issues with the ‘other hay’ category seen in 2007 [26], and makes it easier to compare against similar studies that extend across the entire US [31]. To identify detailed patterns of land cover change, we used transition diagrams to track the “to and from” changes of each individual pixel [28, 30]. Transition diagrams show the complicated pathways that can exist on a changing landscape, and can therefore help identify potential direct land cover change effects [49]. We summarized the data as the net change of a class by taking the difference of the ‘from and to’ changes in order to account for crop rotations (such as corn and soy) (after [24]). Using transition diagrams, we present net changes in land classes as percentage of the total land cover change happening in the region of interest (e.g. southern WI). While transition diagrams provide information on the net change happening over time, we organized our results by year, in order to assess the temporal variability of land cover change in the region. The transition diagrams, one for each region in each state, show the total area that underwent land cover change. Each of the five land classes is represented by an arc with the width of the link connecting two arcs representing land cover change between the corresponding groups as a fraction of total land cover change in that region.

### Potential Sources of Error

The accuracy of this analysis is partially dependent on the datasets used (CDL, SSURGO, and PAD). The CDL thematic land cover dataset is particularly important as we used it to define non-agricultural and agricultural lands. Generally, the agricultural classes have a very high accuracy (80 to 95%) but the non-agricultural classes do not. To account for the low accuracy of non-agricultural lands we combined individual groups with similar spectral signatures together to create a larger group with a higher overall accuracy [50]. We filtered out single pixels in our “Biophysical land Identification” analysis. Extensive sensitivity analysis showed that removing single pixels greatly reduced erroneously classified pixels that were a combination of the 2008 or 2013 CDL dataset and the SSURGO LCC dataset. Since any errors inherent in geospatial data are compounded when multiple datasets are fused together [51], it is important to interpret the results with caution. Focus should be given on the general trends and not exact values. This is also important when comparing between two values, such as a change in marginal open lands between 2008 and 2013. The overall combined change rate that we compute will be lower than the sum of the separate change rates because some changes occurs multiple times in the same pixel. In such a scenario, we take the most recent land cover class as the one represented. The accuracy of our change classification is dependent on that of the underlying CDL. While CDL has producer and user accuracies between 85%-95% for major row crops like corn and soybean, its accuracies are quite a bit lower for specialty crops, fruits and vegetables, and in distinguishing between the subtle spectral signature variations among the various non-agricultural land-cover classes, especially in the grassland category.

## Results

### Biophysical Land Identification

Removing public land area reduced agricultural lands, averaged between 2008 and 2013 in the northern region, by 2.7% and reduced open lands by 13.8%. Our subsequent analysis excludes any reporting of these public lands. Here we present key changes in land cover area for different land groups and site-quality classes for each of the Lake States. Unless otherwise specified, any percentage specified within parentheses represents the relative change in land cover area for a specific scenario.

### Land Cover Change in the Lake States

**Fig 2:**
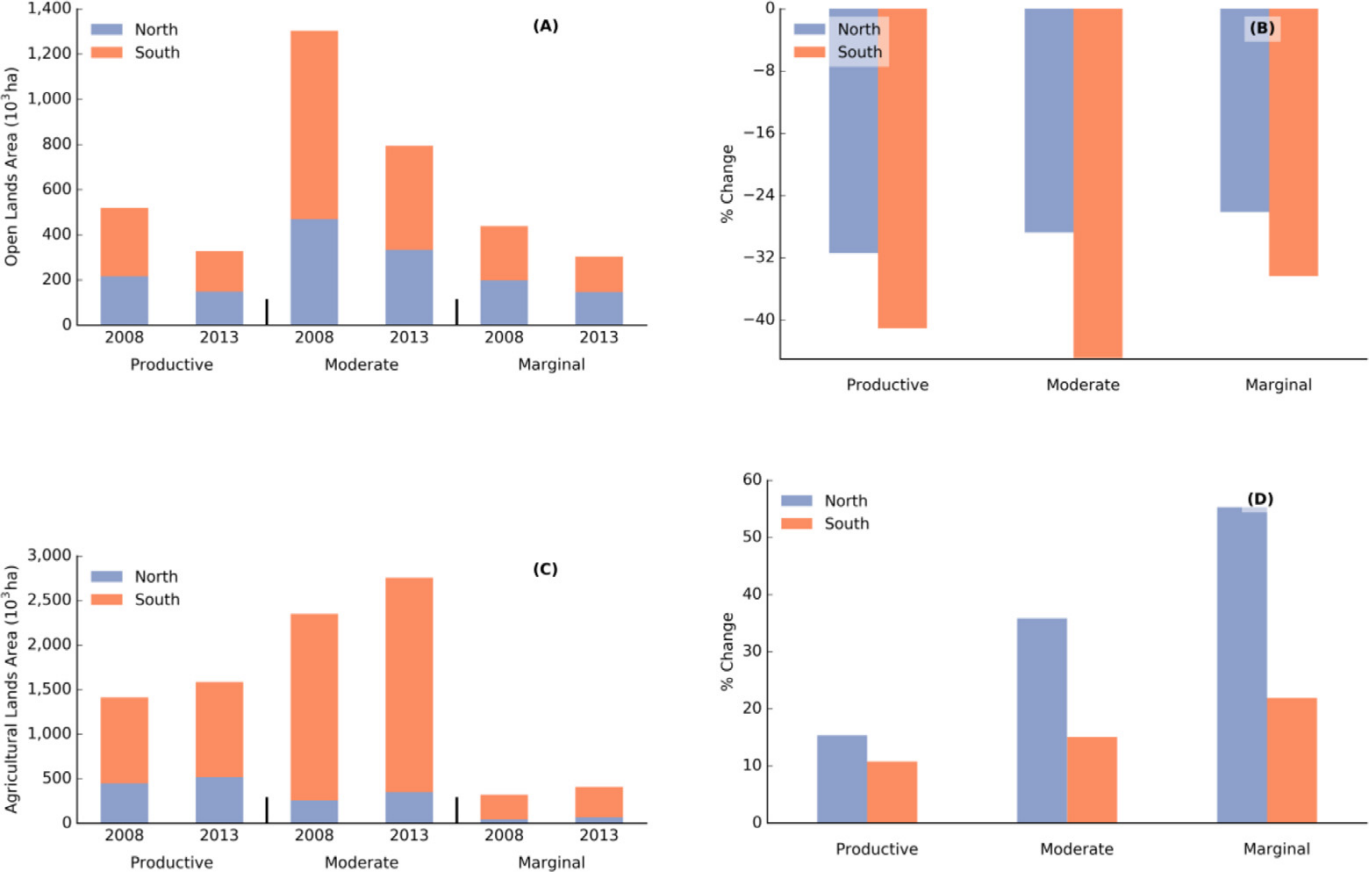
Non-agricultural open lands and agricultural lands along a site quality gradient in 2008 and 2013 in the northern and southern Lake States region. (A) Non-agricultural open land area in 2008 and 2013 in the northern and southern region; (B) the relative change of open lands between 2008 and 2013 in the northern and southern region; (C) agricultural land area in 2008 and 2013 in the northern and southern region; (D) the relative change of agricultural lands between 2008 and 2013 in the northern and southern region. Data shown are in thousands of hectares (A and C) and % change (difference between 2008 and 2013 divided by 2008 * 100). Note the differences in scale for all four graphs.

Across all site-quality classes, non-agricultural open lands decreased in area by 255x10^3^ ha (28.8%) in the north and 581x10^3^ ha (42%) in the south (Fig 2A and 2B). Both absolute and relative decreases in open land area were greater in the south for each site-quality class. Agriculturally marginal open lands in the northern portion of the Lake States decreased from 199x10^3^ ha to147x10^3^ ha between 2008 and 2013, for a decrease of 26%, while in the south, these open lands recorded the most absolute decrease in area from 240x10^3^ ha to 157x10^3^ ha (34%) (Fig 2A and 2B). In the same period, open land area in the productive site-quality class decreased by 68x10^3^ ha (31%) in the north and 124x10^3^ ha in the south (41%). The largest relative change was on moderate lands in the south (45% decrease) (Fig 2B).

**Fig 3:**
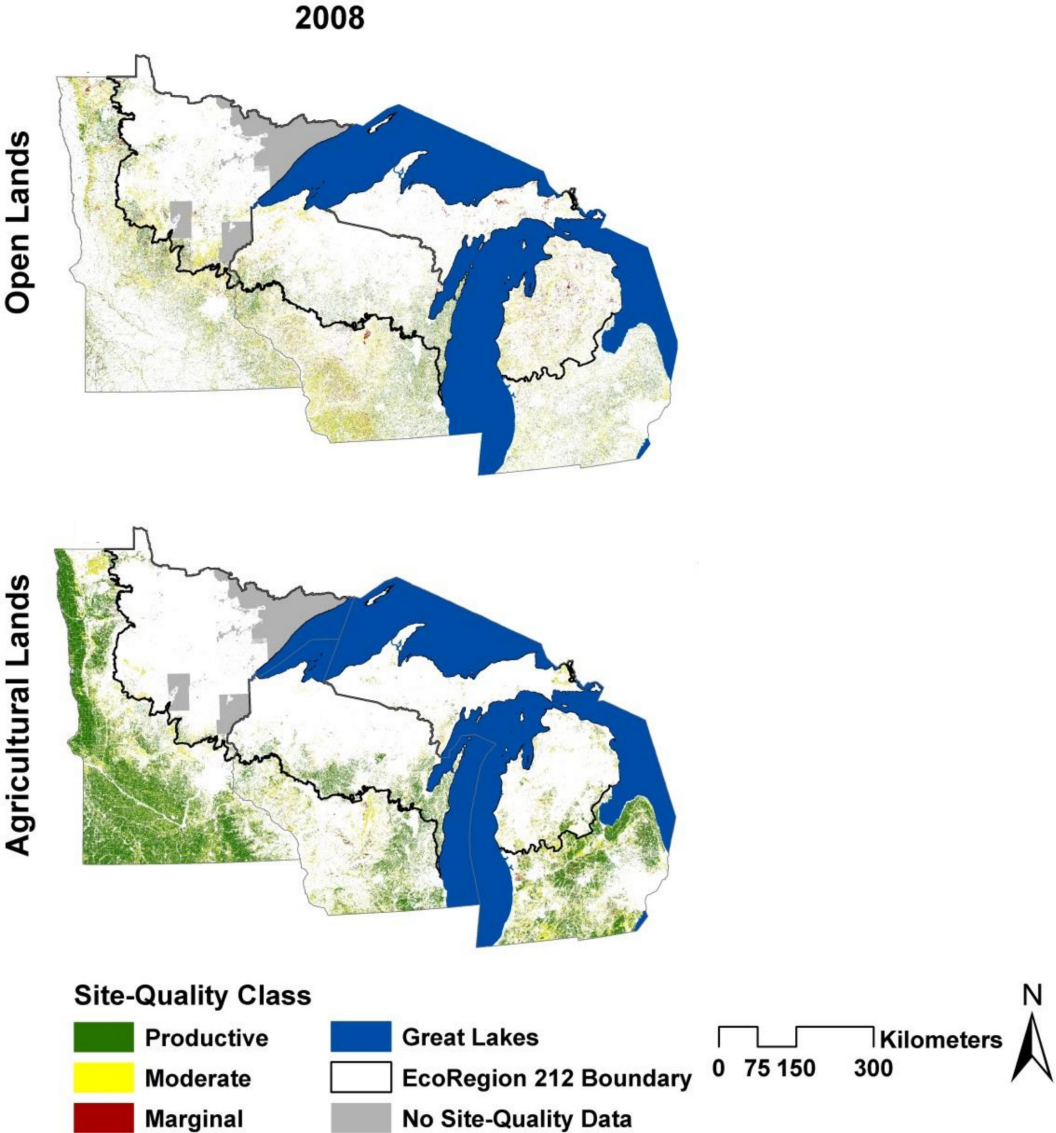
Pixels used in the “Biophysical Land Identification” analysis (Fig 2). The data have been filtered to remove single pixels and areas in public holding. Each pixel has been color-coded to indicate its site-quality class.

In comparison, agricultural lands increased in area for each of our six classes and by 666 x10^3^ ha (16%) overall. The northern and southern Lake States contributed 186x10^3^ ha (25%) and 480x10^3^ ha (14.3%) to this increase respectively (Fig 2C and 2D). The biggest absolute change in agricultural area was observed on moderate lands in the south (315x10^3^ ha), where it increased from 2,091x10^3^ ha to 2,407x10^3^ ha. The largest relative change was on marginal lands in the north, where agricultural lands increased in area by 25x10^3^ ha (from 45x10^3^ ha to 69x10^3^ ha) (Fig 2C and 2D). While the relative change in area was least for the productive agricultural lands, they contributed 173x10^3^ ha or double the increase in area of marginal agricultural lands (Fig 2C). The distribution of these lands is shown in Fig 3.

**Fig 4:**
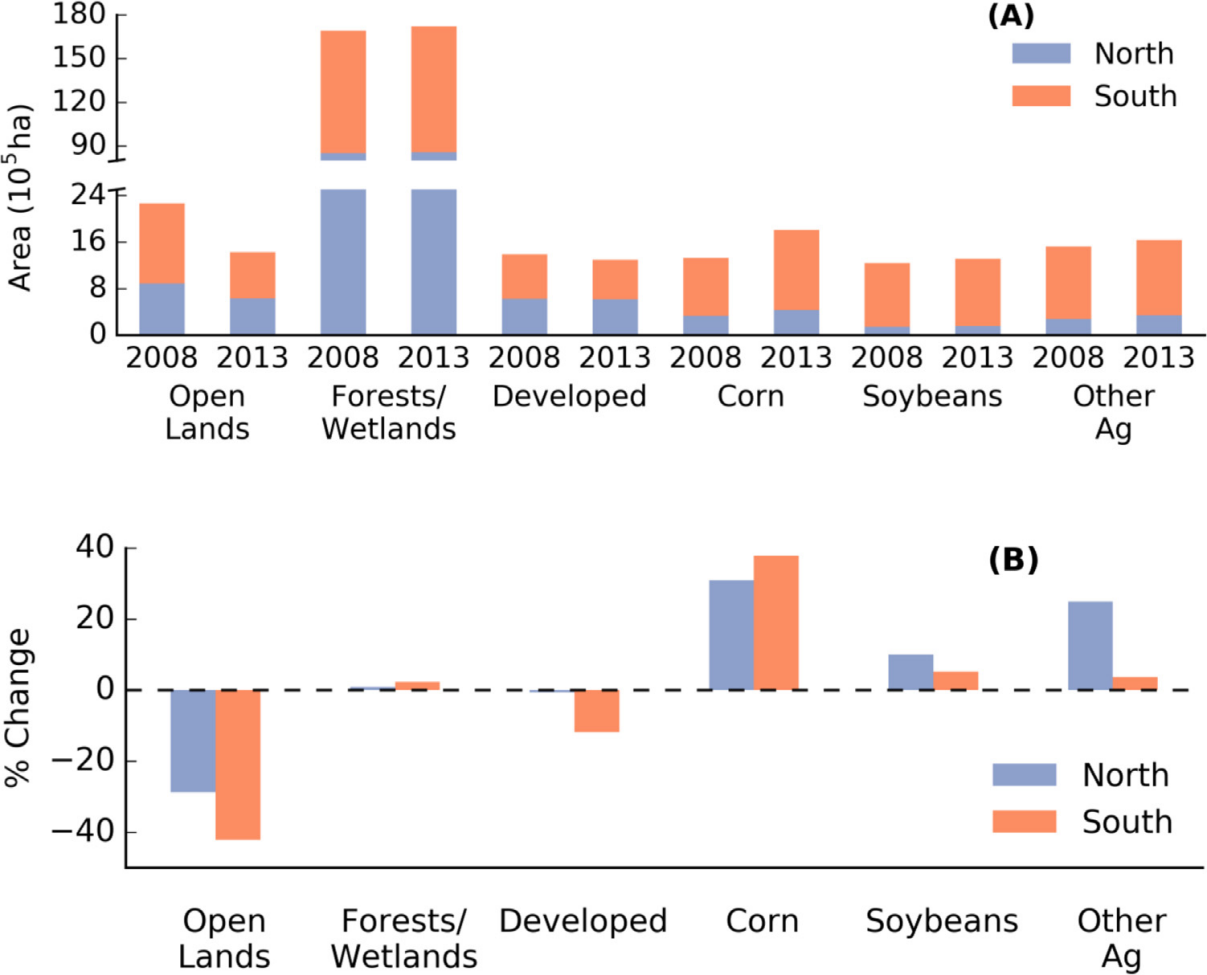
Area of major land use/cover groups from the 2008 and 2013 CDL for the southern and northern Lake States (A). Panel B shows the relative change in area from 2008 to 2013 for each land group in the north and south. Open water and barren lands not included here. Table 1 describes the CDL classes that comprise the groups shown here.

Forests/wetlands comprise the dominant land cover category in the Lake States, covering nearly 70% of the land area of around 24.7x10^6^ ha in 2013 (Figs 1 and 4). Aside from the public lands, nearly half of this land group is within the northern region. A total of 264x10^3^ ha were added to this land group, divided into 73x10^3^ ha and 191x10^3^ ha in the northern and southern lake states respectively (Fig 4). As the next most abundant land group, agriculture increased in area from 4.1 to 4.8x10^6^ ha (17% increase) (Fig 4). Corn and soybeans accounted for approximately 65% of the total agricultural area, while the remaining 35% went to “Other Ag” (Fig 4).

Between 2008 and 2013, the area devoted to corn production increased by 480x10^3^ ha or 36% in the Lake States. Nearly 80% of this increase in area came in the south, with the remaining 103x10^3^ ha of corn area being added in the north. The relative and absolute gains in area for soybean were more modest, with an increase in area of only 15x10^3^ ha (10%) and 57x10^3^ ha (5%) in the north and south Lake States respectively (Fig 4A and 4B). Other agriculture increased by 69x10^3^ ha in the north (25%), and 46x10^3^ ha (4%) in the south (Fig 4A and 4B). Aside from open lands, developed lands were the only other land group to decrease in area between 2008 and 2013. They decreased in area by 94x 10^3^ ha (7%), with the decrease almost exclusively concentrated in the south Lake States. This was smaller in both absolute and relative terms to the loss in open land area that decreased by nearly 836x10^3^ ha (37%).

**Fig 5:**
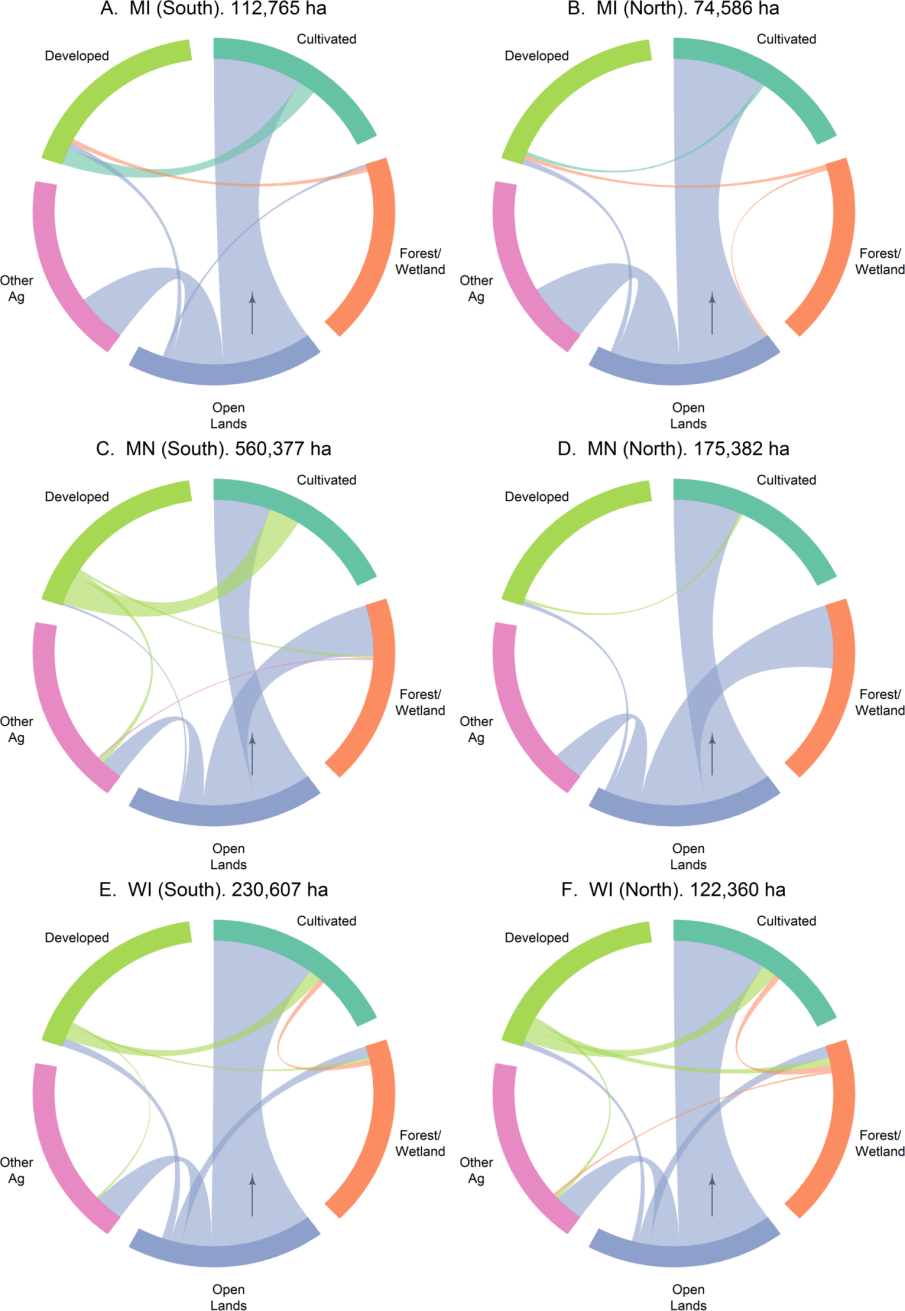
Transition diagrams depicting net changes in major land-cover groups between 2008 and 2013 in the northern and southern regions of each of the Lake States. The numerical estimates above each figure (A–F), represent the total area undergoing land-cover change in each region. The width of the arc represents the percentage of the total that is associated with a specific land-cover change transition e.g. cultivated to developed.

Open land loss dominates land cover change in each of the six regions across the Lake States (Fig 5), accounting for upwards of 50% of the net change in any region. In relative terms, the most open land loss occurred in the northern parts of Minnesota (~75%), with half of the increase ending up in cultivated lands and the other half in forests/wetlands. Within cultivated lands, we break out the ‘other ag’ category, to observe if their dynamics of land cover transitions are any different from the row crops.

**Fig 6:**
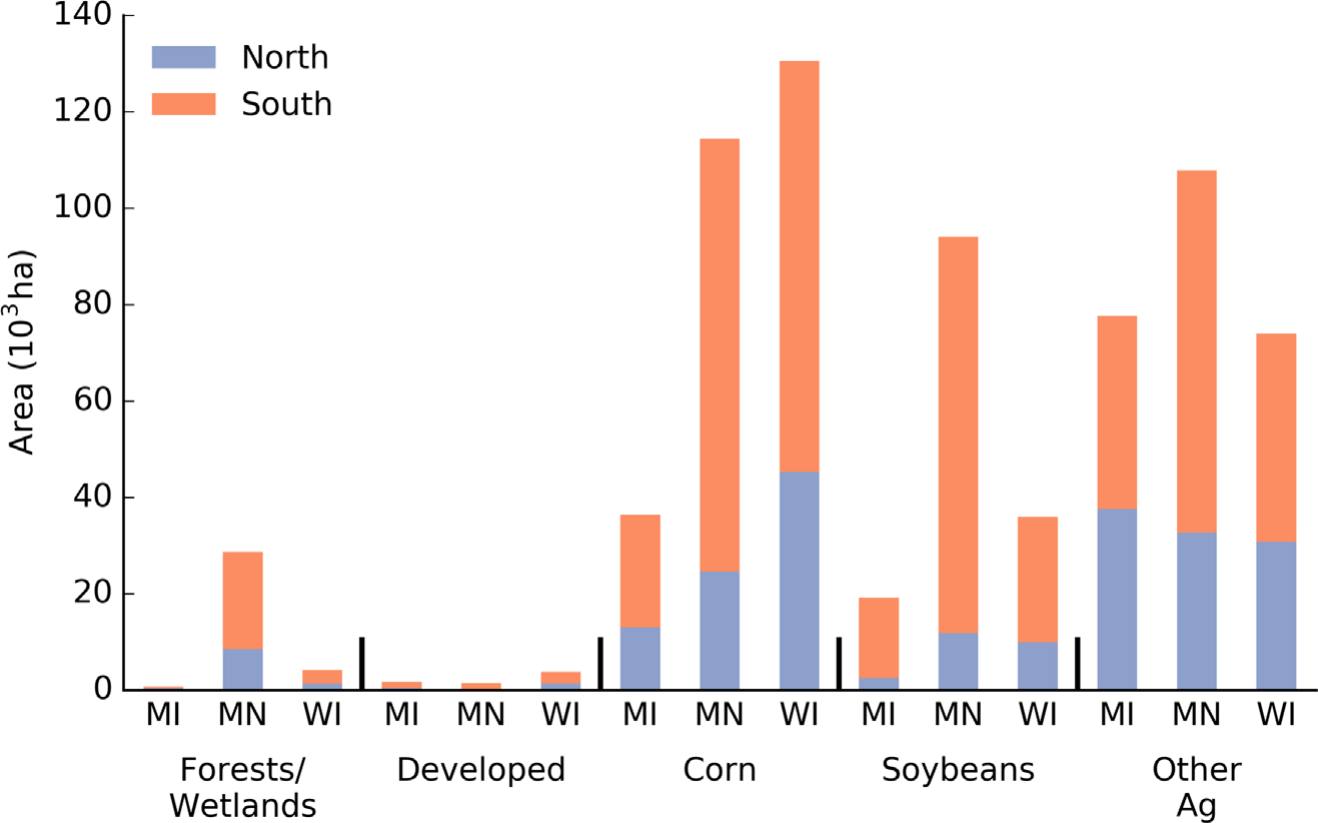
Increase in area of land cover classes as a result of a loss of open lands between 2008 and 2013 in each of the Lake States.

In both the northern and southern Lake States, open lands were mainly lost to cultivation (corn, soybean and other ag). In relative terms, about half of this loss was diverted to the ‘other ag’ component in each region. The absolute amount of this loss varied from 75x10^3^ ha in southern Minnesota to 31x10^3^ ha in northern Wisconsin (Figs 5C, 5F and 6). Within the row crop component of cultivated lands, open land conversions to corn generally greatly exceeded those to soybean, with southern Minnesota being an exception (Fig 6). Conversion to corn (89x10^3^ ha) in southern Minnesota topped the list of open land losses in the region. It was followed closely by southern Wisconsin (85x10^3^ ha to corn) and southern Minnesota (82x10^3^ ha to soybeans) (Fig 6). Aside from southern Minnesota, the magnitude of conversion from open lands to ‘other ag’ was more than that of open lands to soybeans in each region (Fig 6).

Apart from the commonality of open land loss to agriculture, each state showed other minor yet distinct land cover changes. For Michigan, this change was represented by conversion of cultivated lands to developed lands (13x10^3^ ha) and forests/wetlands to developed lands (5x10^3^ ha) (Fig 5A and 5B). In Minnesota, we observed nearly 102x10^3^ ha of developed lands converting to agriculture (Fig 5C and 5D). Wisconsin was the only state where a net positive amount of land converted to agriculture (11x10^3^ ha) (Fig 5E and 5F). From a regional perspective, land cover transitions were both more diverse and greater in area in the southern Lake States.

While land cover transitions necessarily refer to a starting and ending land cover for a specific time period, we also observed land replacements [49] in southern Michigan. Here, land cover change from agricultural lands to developed lands (11x10^3^ ha) is more than offset by a conversion of 57x10^3^ ha of open lands to agricultural lands (Fig 5A). Land replacement can also be seen in Wisconsin, with conversion of developed lands to agricultural lands (27x10^3^ ha) being partly offset by open land conversion to developed lands (15x10^3^ ha) (Fig 5E).

**Fig 7:**
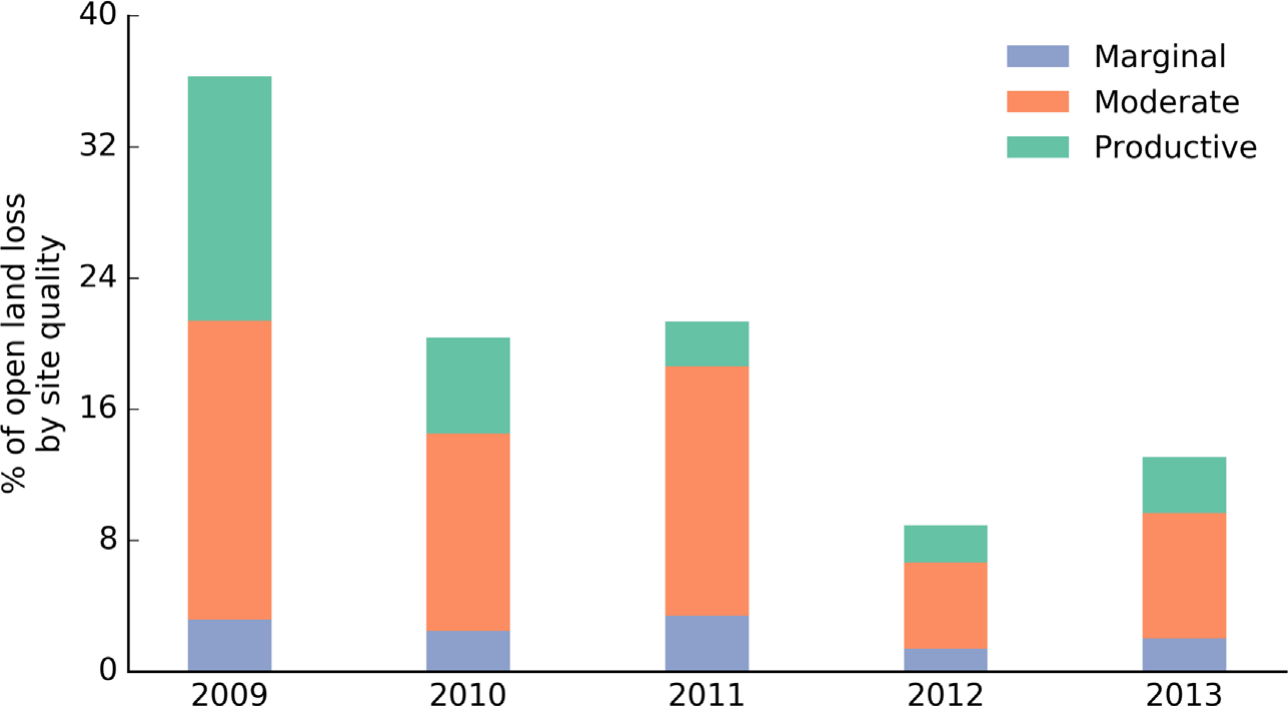
Percentage of open land loss within each site-quality class for each year starting from 2009.

In order to understand the temporal dynamics of open land loss, we examined the area of open lands being converted to other land covers each year. The analysis starts from 2009 since it is the first year for which we produced land cover change information. Around 60% of the total open land loss between 2008 and 2013, happened in the moderate site-quality class, but varied greatly from about 50% in 2009 to about 71% in 2011. Open land loss peaked in 2009, accounting for 37% of the total open land conversion to other land covers in this time period (Fig 7). Concurrently, the percentage of productive lands that were part of this open land loss, decreased from 15% in 2009 to 3% in 2013.

Finally, we used county-specific cellulosic feedstock yields from [23], as representative of open land biomass production potential (Table 2) to estimate potential cellulosic yield of the open lands lost to land cover conversion. This dataset represents the best available spatially explicit estimates of cellulosic feedstock yield estimates for the region, but differs in its consideration of fewer land capability classes (5, 6 and 7) and lack of land cover change signal in the estimates because of their usage of native grasses as cellulosic feedstocks. We estimate that if all of open land acreage available in 2008 in the Lake States was used for cellulosic feedstock production, without factoring relative suitability, we could meet 10% of the Energy Independence and Security Act (EISA) of 2007 mandate for cellulosic feedstock production by 2022. Instead, the open land losses of the last few years have reduced this potential by 37% to 6.4% of the total mandate (Fig C in S1 File).

**Table 2:** Environmental Quality Integrated Climate (EPIC) model simulated biomass and ethanol production potential on open lands in the Lake States in 2008 and 2013 [23]. Results are also presented as a fraction of the Energy Independency and Security (EISA) act of 2007 mandate for ethanol production targets for 2022.

	Open Lands (10^5^xha)			Ethanol (GL yr^-1^)		
	2008	2013	Average Biomass (Mg ha^-1^yr^-1^)	2008	2013	% Change
Michigan	6.8	5.8	10.0	2.6	2.2	-15.2%
Minnesota	9.0	4.1	10.6	3.6	1.6	-54.8%
Wisconsin	6.8	4.4	9.0	2.3	1.5	-35.0%
			Fraction of EISA	10.2%	6.4%	

## Discussion

The presumed potential land base for the sustainable production of perennial cellulosic biomass in the Lake States has been greatly diminished in recent years, largely due to the expansion of agriculture. We found a net loss of 836x10^3^ ha (37%) of non-agricultural open lands to other land covers including row crop agriculture. Most open land loss occurred in northwestern and central Minnesota and throughout southern and central Wisconsin, with relatively smaller 96x10^3^ ha of open land loss in Michigan. The largest relative changes occurred in the southern region of the Lake States, where open lands decreased by 40% during the study period and agriculture increased by 16%. The relative open land losses were more modest in the northern Lake States (29%), but these losses were compensated by greater gains in agricultural lands overall (35%).

On marginal lands in the northern region, agricultural area increased by 55% between 2008 and 2013 on these least suitable areas. This particularly limits options for cellulosic ethanol production in a region with an already lower potentially available land base as compared to the southern Lake States. In addition, these northern lands are climatically limited for agriculture in general, and are largely unsuited to switchgrass [52], and have been targeted for short rotation woody crops (SRWC) of hybrid poplar or willow plantations [48, 53]. This overall loss of open lands reduced the area of land that could potentially be converted to cellulosic feedstocks without inducing indirect land cover change. Our study also underscores the need for inclusion of land cover change effects on GHG balance in modeling studies investigating bioenergy production potential on open lands.

While open land loss was the dominant land cover change trajectory, we did observe two counter-intuitive land cover transitions in the Lake States: a decrease in developed area, and an increase in forests/wetlands area. The former relates to the lower classification accuracy associated with the low-intensity developed/open space lands, a subclass within our developed category (Table 1). These lands have a high proportion of herbaceous vegetation and are therefore often confused with other grass-dominated categories [54]. In the scenario that the urban context of these developed/open space lands has been misrepresented, our results provide a conservative lower bound on the estimate of open land loss in the region. However, it is unlikely that all developed/open space lands in the CDL are actually open lands, since it has been estimated that land cover classifications typically underestimate such low-density developments [55]. The slight increase in forests/wetlands area is happening despite a net forest area decrease in the Lake States [56], and may speak to the effectiveness of local wetland restoration activities [57], as well as lower spectral separability between herbaceous wetlands and other classes. Neither of these issues confounds our larger results.

### Patterns and Drivers of Land Cover Change

The loss of open lands is not unique to the Lake States and has been seen across the U.S. Faber et al (2012) reported a loss of 9.3x10^6^ ha of grasslands, shrublands and wetlands between 2008 and 2011 throughout U.S. due to expansion of principal agricultural crops (corn, soybeans, wheat, cotton and sorghum). They found that corn replaced the largest area of these natural lands (3.3x10^6^ ha), and wheat and soybean accounted for 5x10^6^ ha. Their results are consistent with ours indicating that some of the most intensive conversion of natural lands to corn occurred in southwest WI and central MN (25). Similarly, Wright and Wimberly [24] found widespread land cover conversion from grasslands and wetlands to cultivation in a five-state area of the western and northern Corn Belt (North Dakota, South Dakota, Nebraska, Iowa, and Minnesota). They found a net change of 0.53x10^6^ ha of non-agricultural grasslands to corn and soybean expansion between 2006 and 2011. This changed area is relatively low compared to our results and those from Faber et al [36], but Wright and Wimberly [24] used a fairly aggressive 5x5 majority filter to remove many of the smaller pixels from their analysis. During our filtering analysis, we tried this 5x5 majority filter and found that it removed more than 50% of the pixels that changed from open lands to corn in the Lake States. We felt that this filter was not appropriate for the heterogeneous landscape of our study region, though it may have worked on the open plains.

Open land loss in recent years can also be attributed to decreased enrollment [31], in the USDA’s conservation reserve program (CRP). Stubbs [31] found that the area of land enrolled in the CRP has been declining every year since peak enrollment in 2007, from over 14x10^6^ ha in that year to less than 12x10^6^ ha in 2012, the lowest enrollment since 1989. Lands are enrolled in CRP because they are susceptible to degradation (especially erosion) under agricultural practices and thus the major decline in CRP enrollment threatens landscape health across millions of hectares, and likely results in a disproportionately high negative environmental impact [30].

To avoid competing with food, currently retired lands like those in CRP could be brought back into production. However, if used for growing first generation biofuels, they can not only accrue carbon debt [41] but also add to health costs [58] nutrient runoff and eutrophication [1], and further erosion of biodiversity habitat and other valued services. A recent study by Lark et al. 2015, found rapid expansion of corn belt onto marginal lands and increasing homogenization of the agricultural landscape. Like our study, they used multi-year CDL data for assessing recent shifts in U.S. agriculture. However, they differed in their biophysical land identification. While our study includes alfalfa in the other crop category owing to its low classification accuracy in the CDL [26], Lark et al. explicitly track land cover transitions into and away from alfalfa. They employ a 3x3 majority filter, which while less aggressive than the Wright and Wimberly [24] approach is less suited for the Lake States as compared to our single pixel filter approach. Lark et al. 2015 report a net increase of 168x10^3^ ha in cropland area in the Lake States (2008–2012), compared to a net increase of 690x10^3^ ha estimated in the present study (2008–2013). The USDA census based estimates an increase of 410x10^3^ ha in cropland area in the Lake States (2007–2012). While both studies are on either side of the USDA census estimate, the present study is closer since 2007 was an anomaly in terms of corn plantings which rose 19% over 2006 but then fell 7% in 2008. Our results indicate the need for further study on the appropriate filtering schemes for different regions in the U.S.

Corn and soybeans also expanded onto open lands during the study period. This was expected, as the area of land planted to corn/soybeans in 2012 was greater than any time since 1937 [59]. Increasing ethanol production, rising demand for food and feed throughout the world, and subsidized crop insurance on formerly protected lands, both CRP and other natural ecosystems, is likely responsible for this expansion [30, 36].

Land cover change not only varies spatially but also ebbs and flows annually based on a variety of exogenous factors such as grain prices, drought conditions, market stability etc. On examining the temporal variations of open land loss in the Lake State region, we find that the losses peaked early, with 2009 accounting for nearly two-fifth of the open land loss (Fig 7). While this can be interpreted as land cover change in the region pivoting away from open land loss, it might also signify lower farmer interest in cultivating the remaining open lands as productive open land availability declined after 2009. While it is difficult to predict what might happen in future years, the increase in number of CRP leases expected to expire in the forthcoming decade might contribute to an increase in open land loss rates as well [41].

### Sustainable Biofuels Landscape

The search for beneficial biofuels should focus on the twin objectives of sustainable biofuel feedstocks that do not compete with food and fiber crops and do not induce either direct or indirect land cover change [60]. The greatest benefit from perennial cellulosics could be achieved on lands where there is room to improve ecosystem services, increase carbon sequestration, and reduce GHG emissions. Idle, abandoned, or degraded agricultural lands (which would be categorized as non-agricultural open lands in our classification) fit this definition much more precisely than simply “marginal lands” [61]. With the recent major expansion of agriculture, and with corn/soybean production levels not seen since 1937, it is quite likely that there are very few easily-converted non-agricultural lands still present. The relatively less common open lands in the forested northern Lake States that are climatically less well suited to corn agriculture, were presumed to be potentially available for woody plantations of poplar and willow. Yet these lands have also been converting to row crops. At multiple locations in northern Wisconsin, forest has been cleared and planted to corn (DJM, personal observations) a change that has not occurred significantly for over 100 years [28] because of short growing season and soils of low value for agriculture. Those open lands that were not converted to agriculture were likely intact native ecosystems that are valuable for a number of purposes, including wildlife habitat. Conversion of these lands in the future, even to perennial cellulosic biomass crops, could result in numerous negative environmental consequences [23]. However, non-agricultural lands may not be most sought after by landowners. If the economics of hybrid poplar or switchgrass became more favorable than traditional agricultural crops, then it would seem likely that farmers who would plant perennial cellulosics would rather displace their agricultural crops than convert their non-agricultural open lands [62]. This suggests that energy crops are more likely to compete with agricultural lands than non-agricultural lands, given appropriate incentives [25].

Past and present land-cover mapping and change detection studies have focused primarily on validation rather than uncertainty estimation [63] and it is generally recognized that post-classification land-cover change detection can be problematic [24]. In our study, we focus on reducing uncertainty in our estimation of land-cover change in the U.S. Lake States. We do not delve into the rather more complicated question of uncertainty characterization because of lack of access to field data or error estimates associated with the raw datasets that we use. Since we do not know which pixels are pure, we try to infer that based on the properties of its neighboring pixels. Our approach is consistent with recent literature [24, 25].

The replacement of corn grown on the most marginal lands with perennial cellulosics that are better suited to marginal conditions offers a potential solution to reducing the negative impacts of corn production, and provides a place to establish perennial cellulosics without further degrading the landscape, or displacing food and feed production [53]. This scenario is unlikely, however, as the corn-grain ethanol industry has many large conversion facilities already existing, and the Federal corn ethanol mandate remains in place. It is also not clear that they would be readily converted to handle cellulosic ethanol since the technology to do so at that scale remains undeveloped [11]. Finally, the net GHG emission abatement benefits of perennial cellulosics are not certain and are sensitive to the changes in bacterial and fungal community composition induced by land use change [64].

### Conclusion

The U.S. is currently in a time of massive agricultural expansion, which competes with lands potentially suited for bioenergy feedstocks and affects the ecosystem services provisioned by the native ecosystems. In our evaluation of the magnitude and effects of recent agricultural expansion (2008–2013) of the area of lands potentially suited for bioenergy feedstock production in the Lake States, we find that over 836x10^3^ ha of non-agricultural open lands were already converted to agricultural uses between 2008 and 2013 in the Lake States, a loss of nearly 37%, and attributable largely to an increase in row crop cultivation to satisfy corn ethanol production mandates. The loss of non-agricultural open lands was highest on productive and moderate quality sites but also included marginal sites that are typically less suitable for row crop cultivation, climatically marginal lands in the northern forested region, and were assumed less likely to compete with food production and incur carbon debt [64]. But the large use of corn, a food crop, for biofuel negates this distinction. While the relative loss of open lands is lower in the forest-based economy of the northern area of the Lake States, available lands are also much less than in the southern region. The shrinkage of the assumed potential land base for the sustainable production of cellulosic biomass peaked in 2009 and was driven by the loss of productive open lands in southern Minnesota and Wisconsin. As a result, even if the recently lost open lands were brought into perennial feedstock cultivation, there would be a substantial carbon debt. We conclude that the window of opportunity for establishing a sustainable perennial cellulosic feedstock economy in the Lake States as laid out in Federal policy is rapidly closing, with a series of cascading negative effects on cellulosic potential, unintended land use change, a growing GHG debt, and loss of ecosystem services from formerly natural lands.

## Supporting Information

S1 File Supplementary information for the manuscript. (DOCX)
