# Supplementary material for: Recent Land Use Change to Agriculture in the U.S. Lake States: Impacts on Cellulosic Biomass Potential and Natural Lands
Source: PLoS One. 2016 Feb 11;11(2):e0148566. doi: 10.1371/journal.pone.0148566 (PMC4750971; doi:10.1371/journal.pone.0148566)
Supplement: S1 File — (DOCX) [file pone.0148566.s001.docx]

**Supplementary Information (S1 File)**

**Recent land use change to agriculture in the U.S. Lake States: Impacts on cellulosic biomass potential and natural lands.**

**David J. Mladenoff, Ritvik Sahajpal, Christopher P. Johnson, and David E. Rothstein**


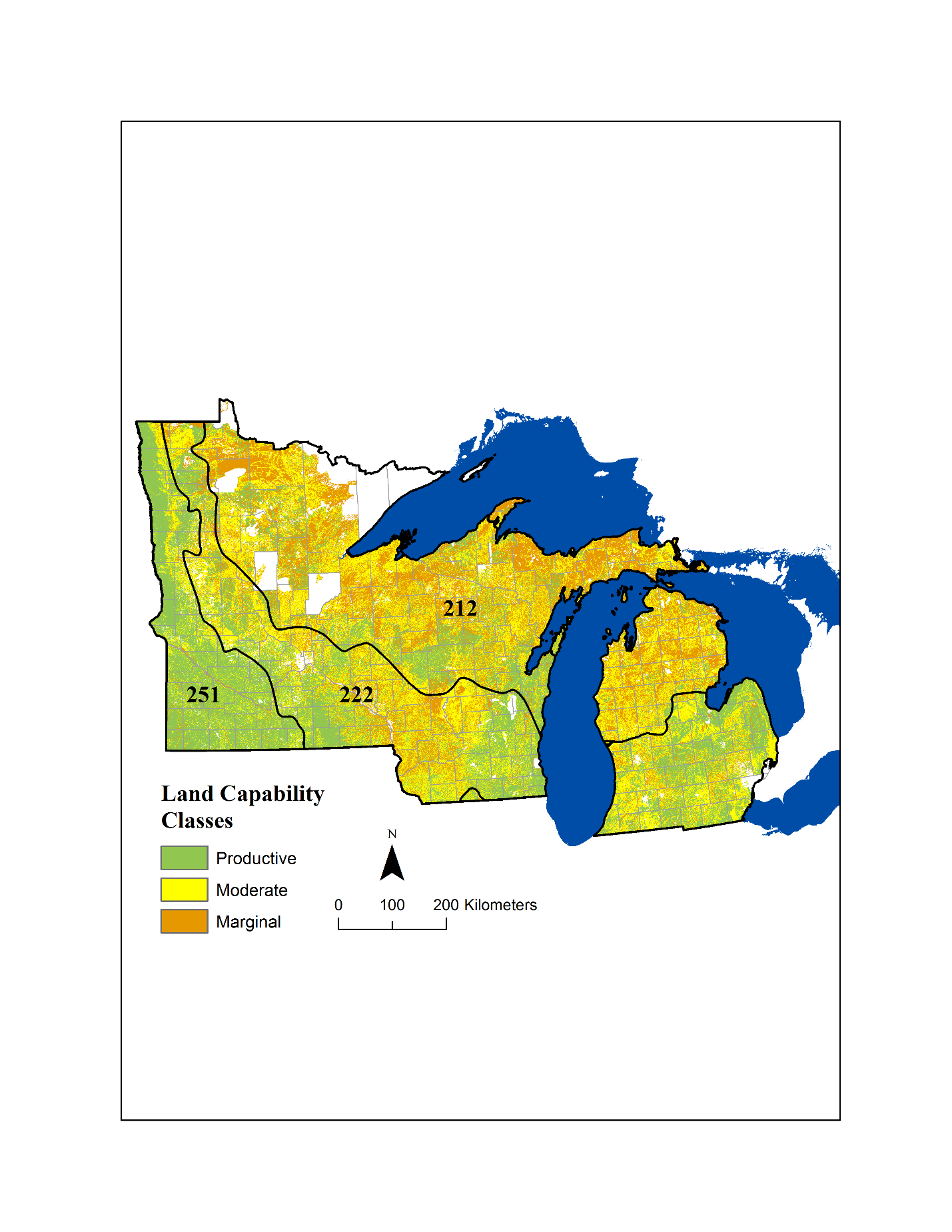


## Figure A. Ecoregion provinces and distribution of land capability classes in the Lake States. Here, we have grouped Ecoregions 251 and 222 together as the “Southern Region” and Ecoregion 212 as the “Northern Region”. Ecoregion 212 is a predominately forested landscape, and therefore particularly suited for SRWC plantations.


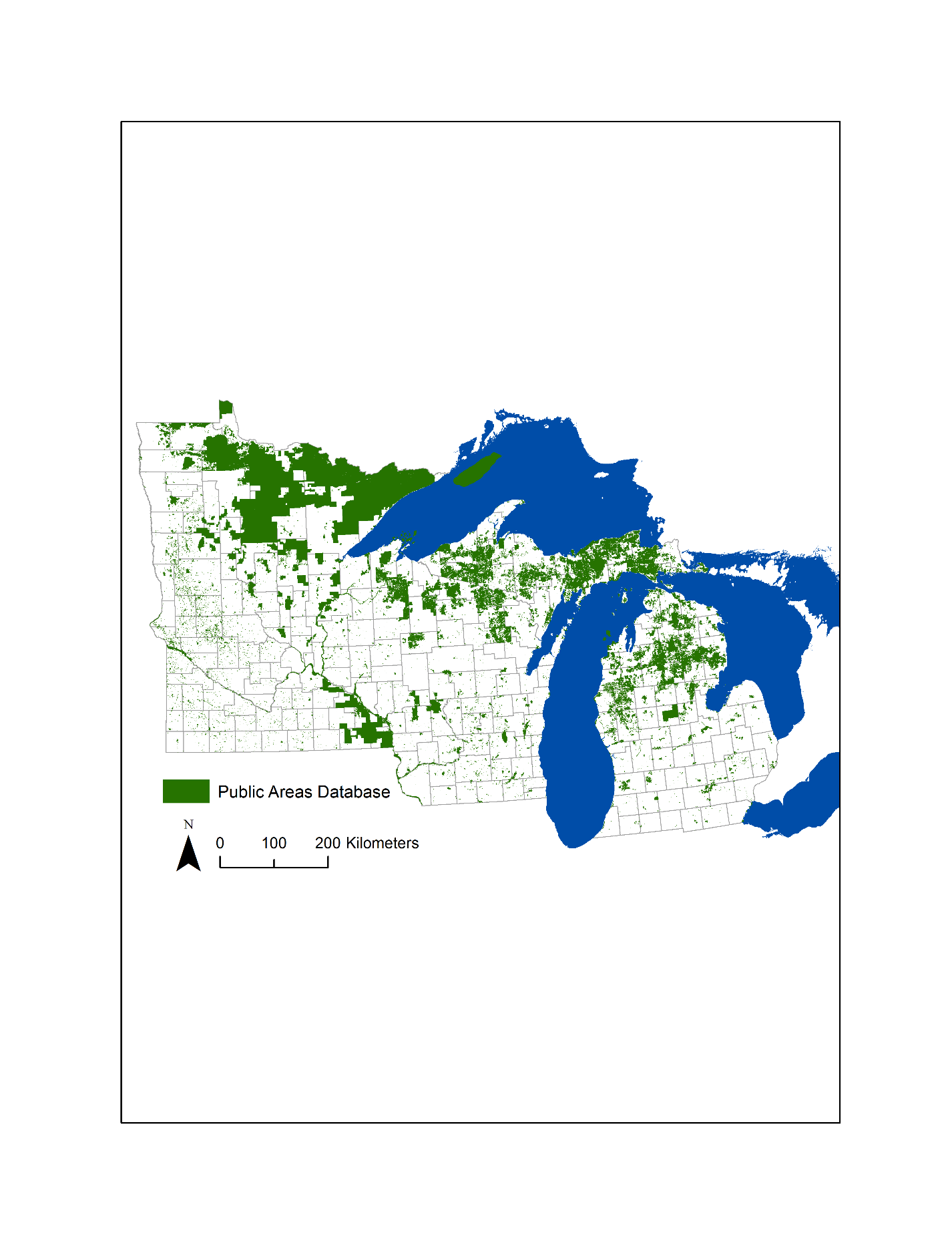


## Figure B. Distribution of public lands in the Lake States.


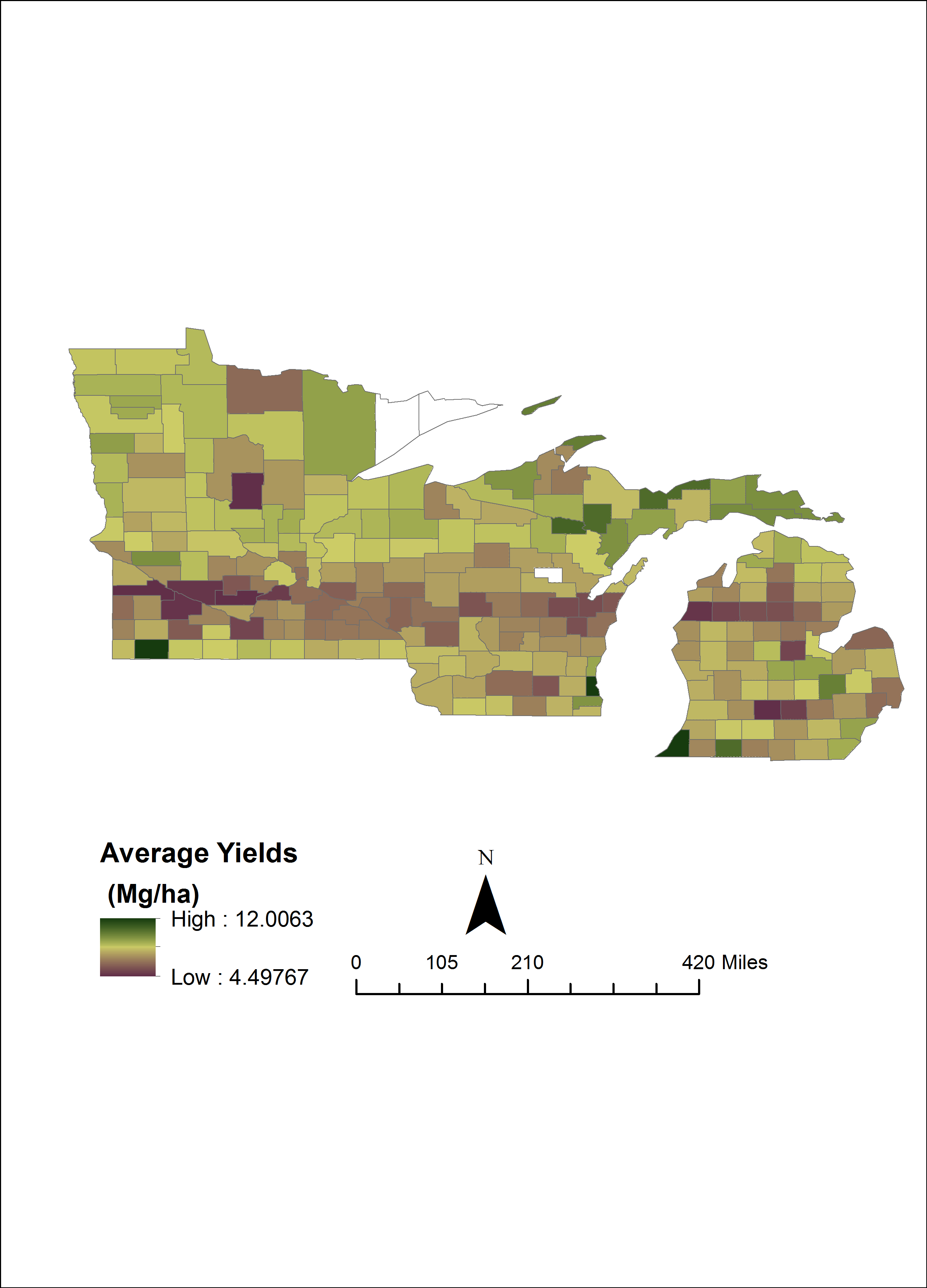


## Figure C. County specific cellulosic feedstock yields on marginal lands in the Lake States based on Gelfand et al. 2013.
